# Predicting chromatin organization using histone marks

**DOI:** 10.1186/s13059-015-0740-z

**Published:** 2015-08-14

**Authors:** Jialiang Huang, Eugenio Marco, Luca Pinello, Guo-Cheng Yuan

**Affiliations:** Department of Biostatistics and Computational Biology, Dana-Farber Cancer Institute, Boston, MA 02215 USA; Department of Biostatistics, Harvard T.H. Chan School of Public Health, Boston, MA 02115 USA; Harvard Stem Cell Institute, Cambridge, MA 02138 USA; Present Address: Editas Medicine, Cambridge, MA 02138 USA

## Abstract

Genome-wide mapping of three dimensional chromatin organization is an important yet technically challenging task. To aid experimental effort and to understand the determinants of long-range chromatin interactions, we have developed a computational model integrating Hi-C and histone mark ChIP-seq data to predict two important features of chromatin organization: chromatin interaction hubs and topologically associated domain (TAD) boundaries. Our model accurately and robustly predicts these features across datasets and cell types. Cell-type specific histone mark information is required for prediction of chromatin interaction hubs but not for TAD boundaries. Our predictions provide a useful guide for the exploration of chromatin organization.

**Electronic supplementary material:**

The online version of this article (doi:10.1186/s13059-015-0740-z) contains supplementary material, which is available to authorized users.

## Background

Chromosomal DNA is packaged into the nucleosomes, each containing an octamer of histone proteins. Histone modifications are known as post-translational modifications at histone tails, such as acetylation, methylation, phosphorylation, and ubiquitination [1]. Genome-wide distribution of histone modifications can be profiled using chromatin immunoprecipitation followed by high-throughput sequencing (ChIP-seq) [2]. Functionally, histone modifications serve as distinct markers for transcriptional regulation and many other biological processes through controlling the accessibility of DNA and recruitment of specific proteins [3–6].

In addition to the nucleosome positioning and histone modifications, the chromatin also undergoes additional layers of compaction through DNA looping and folding, forming complex, dynamic 3D structures. Genome-wide mapping of the 3D chromatin organization and its dynamic changes will provide important insights into the cell-type specific gene regulation and functions of genetic information [7]. A number of technologies, including 3C, 4C, 5C, ChIA-PET and Hi-C, have been developed to experimentally map long-range chromatin interactions [8]. Among these technologies, Hi-C provides the most comprehensive view of genome-wide chromatin interactions [9].

Recently, several Hi-C datasets have been generated and deposited in the public domain [9–15]. Analyses of these data reveal distinct features such as chromatin compartments [9], topologically associated domains (TADs) [10], and chromatin loops [12]. However, it remains difficult and costly to map genome-wide chromatin interactions at high-resolution. In contrast, ChIP-seq experiments can be routinely carried out by many labs at much lower cost, and there is already a large amount of data in the public domain. It has been noted that chromatin interactions are associated with distinct patterns of histone modifications [9, 10, 16, 17], suggesting computational predictions may be a cost-effective approach to guide the interrogation of the global landscape of chromatin interactions.

To this end, we have developed a computational model to predict two important features of chromatin organization: chromatin interaction hubs ("hubs" for short) and TAD boundaries. We define hubs as the genomic loci with frequent chromatin interactions. Intuitively, these hubs serve as the nucleation sites of chromatin looping thereby playing an important role in gene regulation. Our analysis shows that these hubs are highly enriched with previously annotated regulatory regions. We find that both features can be predicted from histone modification patterns with good accuracy, but these patterns differ significantly in terms of predictive marks and cell-type specificity. The predictions are robust across datasets and cell types.

## Results

### Chromatin interaction hubs are enriched with regulatory regions

**Fig. 1:**
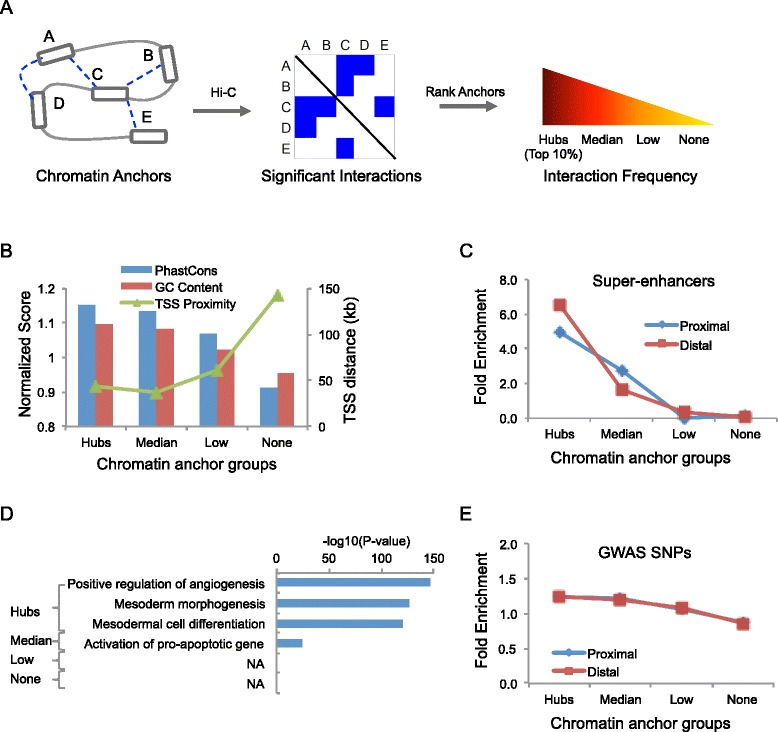
Overview of chromatin interaction hubs. **a** Definition of chromatin interaction hubs. Chromatin anchors are ranked based on the frequency of significant interactions and classified into four group: Hubs, Median, Low, None. **b** DNA sequence of hubs. The average PhastCons conservation score and GC Content ratio (*left-y-axis*) within chromatin anchors is normalized against the genomic background. TSS proximity (*right-y-axis*) is represented by the distance to the closest TSS. **c** Enrichment of the super-enhancers in IMR90 cells. Chromatin anchors in each group are further divided into two subgroups are according the distance to their closest TSS, Proximal (<100 kb) and Distal (> = 100 kb). **d** Functional enrichment analysis using GREAT. **e** Enrichment of the SNPs in GWAS catalog. Chromatin anchors in each group are further divided into two subgroups according the distance to their closest TSS, Proximal (<100 kb) and Distal (> = 100 kb)

We analyzed a public, high-resolution Hi-C dataset by Jin *et al.* [11], obtained from IMR90 cells, a human fetal lung fibroblast cell line. In their study, the Hi-C data was normalized by adapting a method previously developed by Yaffe and Tanay [18] to further incorporate normalized distance and fragment size jointly [11]. Then, by applying a peak calling algorithm, Jin *et al.* identified a total of 1,116,312 statistically significant chromatin interactions among 518,032 chromatin anchors at 5–10 kb resolution by combining multiple consecutive restriction fragments [11]. Based on these significant chromatin interactions, we ranked the chromatin anchors according to interaction frequency and classified them into 4 groups (Fig. 1a and Additional file 1: Figure S1A). The “Hubs” group, containing top 10 % of chromatin anchors; the “None” group (~55 %) contains chromatin anchors without significant interactions; and the rest was divided into two roughly equal-sized groups, named the “Median” group and the “Low” group, respectively.

We focused on the hubs and hypothesized they may play an important role in gene regulation. To gain insights into their biological functions, we began by searching for distinct genetic features. We found that the DNA sequence at the hubs was highly conserved (P = 3.9E-60, Student’s t-test; Fig. 1b) compared with the genomic background. The GC content at these hubs was significantly higher (P-value < 2.2E-16, Student's t-test; Fig. 1b). The hubs tended to be closer to the Transcription Start Sites (TSS), with a median distance of 43 kb, compared to other chromatin anchors (Fig. 1b). We also compared the hub locations with super-enhancers, which were previously shown to play an important role in the control of cell identity and diseases [19], and observed a 5-fold enrichment comparing to the genomic background. Further analysis showed that the enrichment was slightly higher in distal hubs than proximal ones (Fig. 1c). In total, 75 % of super-enhancers overlapped with at least one hub (Additional file 1: Figure S1B). Furthermore, functional enrichment analysis using GREAT [20] showed that genes nearby the hubs were significantly enriched for development-related processes, such as mesoderm morphogenesis (P-value = 1.0E-126) (Fig. 1d). Recently, integrative analysis of 111 reference human epigenomes reveals that tissue-specific regulatory elements are enriched in disease- and trait-associated genetic variants [21]. Thus, we tested whether these hubs were associated with disease associated variants. We found that these hubs were 1.3-fold enriched for the single nucleotide polymorphisms (SNPs) in the genome-wide association studies (GWAS) catalog (Fig. 1e, Methods). Taken together, the above results strongly suggest that the hubs play an important role in the establishment of cell-type specific gene regulatory programs and that genetic variation at these loci may lead to increased risk of diseases.

### Histone marks are highly effective for predicting hubs

**Fig. 2:**
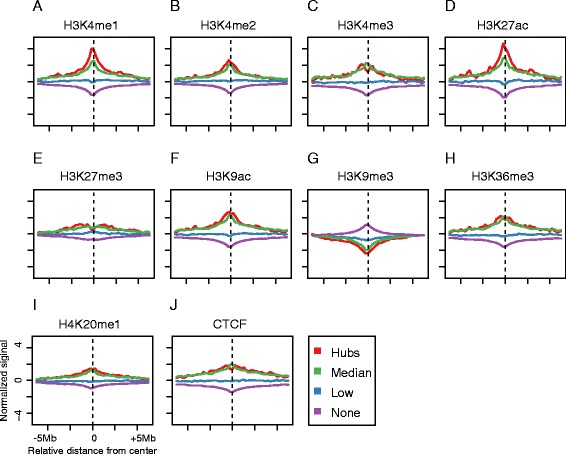
Histone mark signatures of hubs. **a**-**j** The distribution of 9 histone marks and CTCF around the center of chromatin anchors. In each panel, the curves with different color represent the four chromatin anchor groups shown in Fig. 1, Hubs (*red*), Median (*green*), Low (*blue*) and None (*purple*). The normalized signal (*y-axis*) was calculated using the histone mark ChIP-seq signal minus the input signal

**Fig. 3:**
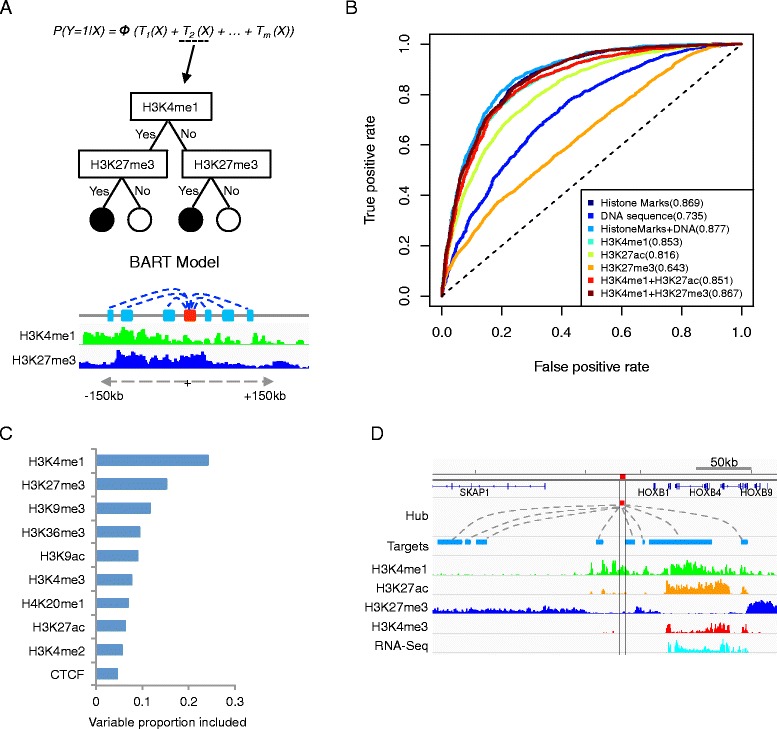
Prediction of Jin2013 hubs in IMR90 cells. **a** Schematic of the BART model. **b** Prediction accuracy using various features. The ROC curves correspond to the testing data. AUC scores are shown in parentheses. "Histone Marks" represents the combination all of histone marks and CTCF, while "DNA sequence" represents the combination of PhastCons conservation score, TSS proximity and GC content. **c** Variable selection in BART model. The x-axis represents the usage frequency of each variable in the BART model. **d** Genome browser snapshot at a hub adjacent to the HOXB gene cluster

To characterize the epigenetic determinants of hubs, we examined the spatial patterns of CTCF and 9 histone marks adjacent to each chromatin anchor (Methods) (Fig. 2). The most distinct features were the elevated levels of H3K4me1 and H3K27ac, both are well-known markers for enhancer elements, around the center of the hubs compared to other chromatin anchors. In addition, there were also significant albeit weaker differences among several other histone marks. In order to systematically investigate how well these hubs could be predicted from the combination of multiple histone marks, we built a Bayesian Additive Regression Trees (BART) model to classify chromatin anchors based on histone mark ChIP-seq data alone. BART is a Bayesian "sum-of-trees" model [22], averaging results from an ensemble of regression trees (Fig. 3a). Previous studies have shown that BART is effective in modeling various computational biology problems [23].

For each hub, we summarized the local pattern for each histone mark by averaging the sequence reads over a 300 kb window (about twice the average distance between an anchor and its target site [11]) centered at the hub location. These summary scores were used as input for model prediction. The Negatives set was chosen to be the chromatin anchors with fewest but non-zero interactions and had the same size as the set of hubs (Positives set). The reason for excluding chromatin anchors associated with no detectable interactions was to remove the bias toward mappable genome and GC-rich sequences. To avoid over-fitting, we divided the Positives and Negatives sets into two equal subsets used for model training and testing, respectively. The prediction accuracy was assessed using the testing subset.

We found that the hubs were well predicted using histone marks (Area Under the Curve, or AUC = 0.869, Fig. 3b), whereas adding certain DNA sequence information, such as PhastCons conservation score [24], TSS proximity and GC content did not further improve the prediction accuracy significantly (Fig 3b and Additional file 1: Figure S1C). Among all the marks included in our model, H3K4me1 was the most informative predictor (Fig. 3b-c, Additional file 1: Figure S1D). Of note, H3K27me3 was selected as the second most frequently used predictor even though it did not show significant enrichment at the hubs (Fig. 3b, Additional file 1: Figure S1C). To test if this was an artifact, we compared the performance of a reduced model with H3K4me1 and H3K27me3 only with an alternative model with H3K4me1 and H3K27ac only, and found that combination of H3K4me1 and H3K27me3 was more effective (Fig. 3b), suggesting that H3K27me3 provides non-redundant predictive information. This conclusion was further supported by visualization. For example, there was a hub between the gene SKAP1 and the HOXB genes cluster and it interacted with 8 different targets (Fig. 3d). Two of the targets corresponded to H3K27ac peaks, but they also overlapped with H3K4me1 peaks and therefore did not provide additional information. In comparison, four of the other targets around gene SKAP1 were enriched with H3K27me3 but not H3K4me1. Therefore, this hub could not be predicted without using information from H3K27me3.

### Hubs prediction using histone marks is robust across datasets and cell types

**Fig. 4:**
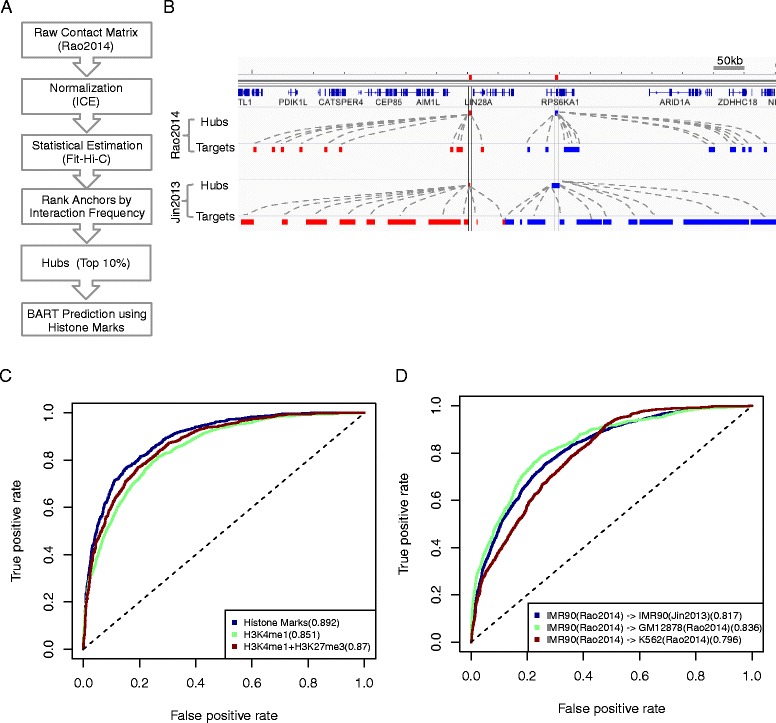
Analysis of the Rao2014 dataset. **a** Workflow for identifying hubs from the raw interaction matrix. **b** Comparison between the Rao2014 and Jin2013 datasets. Genome browser snapshots showing two hubs adjacent to the LIN28A locus (*indicated by red and blue respectively*) and their associated targets in each dataset are shown. **c** Prediction accuracy for the Rao2014 IMR90 hubs. The ROC curves correspond to the testing data. AUC scores are shown in parentheses. **d** Prediction accuracy for applying the Rao2014 IMR90 model to predict hubs in other datasets (Jin2013) or cell-types (GM12872(Rao2014) and K562 (Rao2014)). The ROC curves correspond to the testing data. AUC scores are shown in parentheses

To test the robustness of our prediction, we repeated our analysis on a recently published Hi-C dataset with higher spatial resolution in multiple cell-types [12]. To identify hubs from this dataset, we first normalized the raw interaction matrix (at 5 kb resolution) using the ICE (Iterative Correction and Eigenvector Decomposition) algorithm [25]. Then we identified statistically significant chromatin interactions by using Fit-Hi-C [26] (Methods). We ranked the 5 kb segments by the interaction frequency and defined the hubs as the top 10 % segments (Fig. 4a, Additional file 1: Figure S2A), and referred to this set as the Rao2014 hubs in order to distinguish it from the set of hubs defined from ref. 11 (referred to as the Jin2013 hubs). Despite the difference in experimental protocols, these two sets of hubs overlapped quite substantially. About 60 % of the Rao2014 hubs overlapped with the Jin2013 hubs. For example, the chromatin interaction profiles identified from these two datasets were very similar at the LIN28A locus, and the hub locations were nearly identical (Fig. 4b).

To evaluate the robustness of our computational predictions, we used the aforementioned strategy to classify the Rao2014 hubs for the IMR90 cells and compared the results we obtained from the Jin2013 hubs. As before, the prediction accuracy was quite high (AUC = 0.892) (Fig. 4c, Additional file 1: Figure S2B). Of note, H3K4me1 and H3K27me3, the most informative predictors identified by analyzing the Jin2013 dataset, were also highly predictive for the Rao2014 dataset (AUC = 0.87). In addition, the BART model trained using hubs from Rao2014 well predicted the hubs in Jin2013 (AUC = 0.817) (Fig. 4d), suggesting the model performance could not be attributed to platform-specific artifacts.

Since our ultimate goal is to use histone mark based predictions to guide chromatin interaction profiling, we tested whether our model developed based on the IMR90 dataset was useful for prediction of chromatin interaction hubs from a different cell-type. We applied this model to predict hubs in two different cell-types: GM12878 and K562, using the cell-type specific histone mark data as input. In both cases, the prediction accuracy was good (AUC = 0.836 for GM12878; and AUC = 0.796 for K562) (Fig. 4d). Taken together, these analyses strongly suggest that our model is robust and provides a useful guide for identifying cell-type specific chromatin interaction hubs.

### Predict TAD boundaries using histone marks

**Fig. 5:**
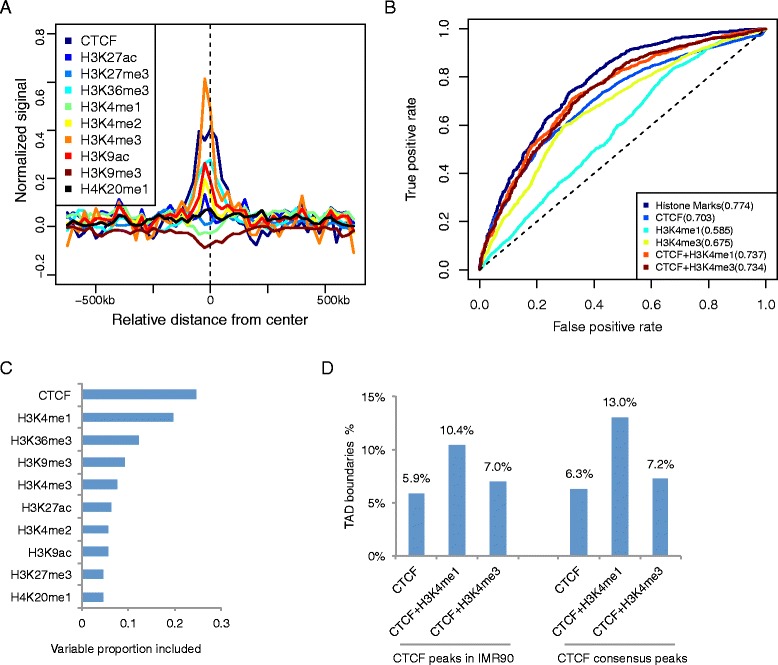
Prediction of TAD boundaries in IMR90 cells. **a** The distribution of various histone marks around TAD boundaries. **b** Prediction accuracy using various features. The ROC curves correspond to the testing data. AUC scores are shown in parentheses. **c** Variable selection in BART model. The x-axis represents the usage frequency of each variable in the BART model. **d** Fraction of CTCF peaks (and filtered subsets) that overlap with TAD boundaries in IMR90 cells. Consensus peaks are defined as those appearing in all 9 cell types

TAD is another important feature in chromatin interactions [10, 27]. Previous studies [10, 12] have shown that distinct patterns of histone marks around TAD boundaries (also see Fig. 5a), but it remains unclear to what extent the boundaries can be predicted by combination of multiple histone marks. To systematically address this question, we applied our modeling approach to predict TAD boundaries by using histone marks. Specifically, we obtained 2,208 TAD boundaries in IMR90 cells identified by Dixon *et al.* [10]. As a negative control, we randomly selected a set with the same size of non-boundary genomic loci with similar interaction frequency. Compared with hubs prediction, we obtained less accurate performance for predicting TAD boundaries using histone marks (AUC = 0.774, Fig. 5b), which might be in part due to the coarser resolution of TAD boundaries. Our model identified CTCF as the most informative predictor (Fig. 5b-c, Additional file 1: Figure S3), which was consistent with the well-known role of CTCF in mediating chromatin interaction sites [8, 10]. However, CTCF plays many different roles in a context dependent manner, and the distribution of CTCF alone is insufficient for predicting chromatin domain boundaries. Consistent with this observation, the performance of using CTCF as the single predictor in our model showed significantly reduced prediction accuracy (AUC = 0.703, Fig. 5b). We found that H3K4me1 was the second most used predictor in our model (Fig. 5c). This observation was somewhat surprising because H3K4me3 was the second most enriched mark at TAD boundaries (Fig. 5a); however, the usage of H3K4me3 in our model was less frequent compared to H3K4me1. We reasoned that the discrepancy might be due to the redundancy between H3K4me3 and CTCF. To test whether H3K4me1 was indeed more useful than H3K4me3 in selecting the TAD boundary associated CTCF sites, we compared the performance of model by using CTCF + H3K4me1 and by using CTCF + H3K4me3, we found that the former indeed had more prediction power (Fig. 5b). Furthermore, we used a simpler approach using the peak information alone (Methods). Out of a total of 26,269 CTCF peaks in IMR90 cells, only 5.9 % overlapped with at least one TAD boundary. This relatively low precision might be in part due to the stringent threshold used for identifying the most distinct TADs. For comparison, combining CTCF peaks and negative H3K4me1 peaks (H3K4me1 was depleted at TAD boundaries) substantially increased the precision to 10.4 %, whereas combining CTCF and H3K4me3 peaks only resulted in a modest improvement to 7.0 % (Fig. 5d). These results suggest that lack of H3K4me1 is indeed a significant signature for TAD boundaries.

**Fig. 6:**
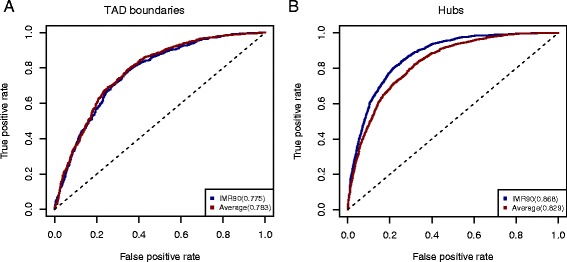
Cell-type specificity of predictions. **a** Comparison of the prediction accuracy of TAD boundaries by using cell-type specific and average histone mark data. The average data were computed based on 8 cell-types other than IMR90. **b** Comparison of the prediction accuracy of hubs by using cell-type specific and average histone mark data. The average data were computed based on 8 cell-types other than IMR90

To test whether cell-type specific histone modification profiles were needed for prediction of TAD boundaries, we obtained ChIP-seq data in 8 other cell types (GM12878, H1HESC, HMEC, HSMM, HUVEC, K562, NHEK, NHLF), and used the average profile as input of the BART model (Methods). Despite the lack of data in IMR90 cells, the prediction performance was almost indistinguishable (Fig 6a), thereby supporting our hypothesis. Similarly, the precision of using the CTCF consensus peaks slightly better than using the IMR90 specific CTCF peaks (Methods, Fig. 5d). This result is consistent with the previous observation that the TAD structure is stable across cell-types [10, 28]. For comparison, we applied a similar analysis to predict the chromatin interaction hubs, and found that the cell-type specific ChIP-seq data was needed to obtain good prediction accuracy (Fig. 6b). Taken together, these results provide new insights into the cell-type specific differences between TAD boundaries and hubs.

## Discussion

Genome-wide exploration of the 3D chromatin organization remains a major challenge. Here we develop a computational approach to use widely accessible ChIP-seq data to predict chromatin interaction hubs and TAD boundaries. In both cases, our models result in reasonable prediction accuracy, supporting the validity of this approach. Using computational modeling, we identified distinctive combinatorial histone patterns between chromatin interaction hubs and regions with few interactions, and between TAD boundaries and internal domains. This information has advanced our understanding of the determinants of chromatin organization, leading to the hypothesis that these combinatorial patterns may be involved in mediating chromatin interactions. This hypothesis can now be tested experimentally, for instance by removal of characteristic histone marks via the CRISPR-Cas9 system.

The concept of chromatin interaction hubs is not new. For example, this has been discussed in a previous study of Pol II mediated chromatin interactions [29]. While previous studies have only focused on specific subsets of chromatin interactions, our current work provides an unbiased and genome-wide view of chromatin organization. It is somewhat unexpected that in this broader context the hubs remain highly enriched with regulatory elements. During the preparation of this manuscript, it came to our attention that another group used a similar approach to link dynamic change of histone modification patterns with chromatin interactions [13]. In that study, H3K4me1 was found to be the most informative predictor for the changes of chromatin interaction frequency, which is consistent with our current analysis. On the other hand, there are significant differences between that study and our work. Aside from the differences in our prediction outcomes, we also went further in investigating the combinatorial patterns of histone marks, and identified H3K27me3 as an additional informative mark for chromatin interaction hubs. Furthermore, we showed that TAD boundaries could be predicted without using cell-type specific histone modification information, which was in contrast with hubs. These results provide new insights into the mechanisms for 3D chromatin structure maintenance.

## Conclusions

We define hubs and show that they mark critical regulatory regions essential in human development and disease. Histone marks are highly effective in predicting hubs and TAD boundaries. H3K4me1 is the most informative predictor for hubs, whereas CTCF is the most informative predictor for TAD boundaries. Combination of multiple histone marks significantly improves the prediction accuracy. We find that prediction of hubs, but not TAD boundaries, requires cell-type specific histone modification information. Our model is robust across datasets. More importantly, we show that the model built from one cell-type can be used to predict the chromatin organization in other cell-types. Our computational approach provides a useful tool for guided exploration of the 3D chromatin organization.

## Materials and methods

### Data availability

The Hi-C data in IMR90 cells for defining hubs was obtained from Jin *et al.* [11], which is available at Gene Expression Omnibus (GEO) with accession number GSE43070. Two files were downloaded from the supplementary data. The file “SuppData3_all_anchors.txt” contains the locations of all 518,032 anchors covering every HindIII fragment in the human genome, while the file “SuppData4_target_of_all_anchors.txt” contains the location of all 1,116,312 significant chromatin interactions. The Hi-C data for TAD boundaries prediction was obtained from Dixon, *et al.* [10], which is available at GEO with accession number GSE35156. The file “Table S4 - Boundaries in mESC, mouse cortex, hESC, IMR90” was downloaded from the supplementary data. The list containing 2,208 TAD boundaries in IMR90 cells was used in our study. The ChIP-seq data of CTCF and 9 histone marks (H3K27ac, H3K27me3, H3K36me3, H3K4me1, H3K4me2, H3K4me3, H3K9ac, H3K9me3, H4K20me1) in IMR90 cells were obtained from NIH Roadmap Epigenome Project [30, 31]. ChIP-seq data of CTCF and 8 histone marks (H3K4me1, H3K4me2, H3K4me3, H3K9ac, H3K27ac, H3K27me3, H3K36me3, H4K20me1) in 8 cell types (GM12878, H1HESC, HMEC, HSMM, HUVEC, K562, NHEK, NHLF) were obtained from ENCODE [32, 33]. All the ChIP-seq data mentioned were aligned to hg18 using Bowtie [34] with default parameter setting. Replicate data were merged if available. RNA-Seq data in IMR90 cells were downloaded from Jin *et al.* [11].

### Identify significant chromatin interactions from Rao2014 dataset

The high-resolution, intra-chromosomal raw interaction matrix in three cell types (IMR90, GM12878_combined and K562) at 5 kb-resolution were downloaded from GEO with accession number GSE63525. To remove the various forms of biases [25, 35] in the raw interaction matrix, we normalized it by using the ICE algorithm [25], as implemented in the Hi-Corrector package [36]. Then we used Fit-Hi-C [26] to identify statistically significant intra-chromosomal interactions, using the parameters -U = 2000000, -L = 10000, with the threshold of FDR = 0.05.

### DNA sequence conservation score

DNA sequence conservation was evaluated by using the 44-way multiple alignment PhastCons score, which was downloaded from [24, 37]. The average conservation score over a 300 kb window was calculated for each chromatin anchor.

### GWAS catalog SNPs enrichment

The SNPs curated in NHGRI GWAS Catalog [38] were downloaded through the UCSC Table Browser [39]. We expanded the GWAS SNPs to include SNPs in strong linkage disequilibrium (LD) using SNAP [40] and perform the enrichment using the expanded set. To remove length associated artifacts, we used a 5 kb window around the center to represent each anchor for enrichment analysis. An anchor is determined to be hit by GWAS SNPs if there is at least one SNP located in the 5 kb window, which was calculated by Bedtools [41]. For each chromatin anchor group, the fold enrichment over genome background was defined as (m/n)/(M/N), where m and M represent the number of within-group and genome-wide SNPs respectively, and n and N represent the number of within-group and genome-wide chromatin anchors respectively.

### Super-enhancer enrichment

The super-enhancers in IMR90 cells were obtained from Hnisz *et al*. [19]. The overlap between the 5 kb window of chromatin anchors with super-enhancers was defined as those sharing at least 1 bp, which was calculated by using Bedtools *intersect* [41]. Fold enrichment analysis of super-enhancers was done as for GWAS SNPs.

### BART model

The BART model consists of three parts: a sum-of-trees model, a set of priors for the structure and the leaf parameters, and a likelihood function for the terminal nodes [42]. For the binary classification problem, the BART model can be expressed as [22]:

$$ P\left(Y=1\Big|X\right) = \Phi\ \left({T}_1(X) + {T}_2(X) + \dots + {T}_m(X)\right) $$

where X represents the histone mark summary scores, Y represents the classification outcome (1 for hub; and 0 otherwise), *T*_*i*_*'*s represent the *i*-th regression tree, Φ denotes the cumulative density function of the standard normal distribution. BART also reports the usage frequency of each predicting variable, which is used as the basis for selecting most informative predictors. We built the BART model using the R package “bartMachine” [22] with default parameters. We also varied the model parameter values, such as different threshold of interactions frequency to define Hubs or different BART parameters, and repeated the prediction analysis. We found that the prediction performance was only slightly affected (Additional file 1: Figure S1E-F). The R code to run BART model for predicting chromatin interaction hubs using histone marks information is available in [43].

### Prediction of TAD boundaries using CTCF peaks

All CTCF and histone mark peaks were called using MACS [44], with a stringent p-value threshold 1.0E-10. To remove length associated artifacts, we used a 250 bp window, the median length of CTCF peaks, around the summit to represent each CTCF peak. The H3K4me3 and H3K4me1 peaks were identified similarly, with the exception that we only considered the negative peaks for H3K4me1 because it was depleted at TAD boundary sites. To obtain a consensus set of CTCF peaks, we obtained CTCF ChIP-seq data in 8 additional cell-types and analyzed as described above. The subset of CTCF peaks that appeared in all 9 cell lines was selected as the consensus peaks.

## Additional file

Additional file 1: Figure S1. Prediction of Jin2013 hubs. (**A**) Distribution of chromatin anchors interactions frequency. Top 10 % are defined as hubs. (**B**) Percentage of super-enhancers covered by hubs. (**C**) Prediction accuracy using DNA sequence genetic features, including PhastCons conservation score, TSS proximity and GC content. AUC scores are shown in parentheses. (**D**) Prediction accuracy using individual histone marks. AUC scores are shown in parentheses. (**E**) Hubs prediction performance for hubs defined using different thresholds of interactions frequency. (**F**) Hubs prediction performance with various number of trees. **Figure S2.** Prediction of Rao2014 hubs. (**A**) Distribution of chromatin anchors interactions frequency. Top 10 % are defined as hubs. (**B**) Prediction accuracy using individual histone marks. AUC scores are shown in parentheses. **Figure S3.** TAD boundary prediction accuracy using individual histone marks. (PDF 1896 kb)

## Abbreviations

TAD: Topologically Associated Domains
SNPs: Single Nucleotide Polymorphisms
GWAS: Genome-Wide Association Study
ChIP-seq: Chromatin immune-precipitation followed by high-throughput sequencing
3C: Chromosome Conformation Capture
4C: Circularized Chromosome Conformation Capture
5C: Chromosome Conformation Capture Carbon Copy
ChIA-PET: Chromatin Interaction Analysis by Paired-End Tag sequencing
Hi-C: Genome conformation capture
TSS: Transcription Start Site
BART: Bayesian Additive Regression Trees
ROC: Receiver Operating Characteristic
AUC: Area Under the Curve
ICE: Iterative Correction and Eigenvector Decomposition algorithm
GEO: Gene Expression Omnibus
